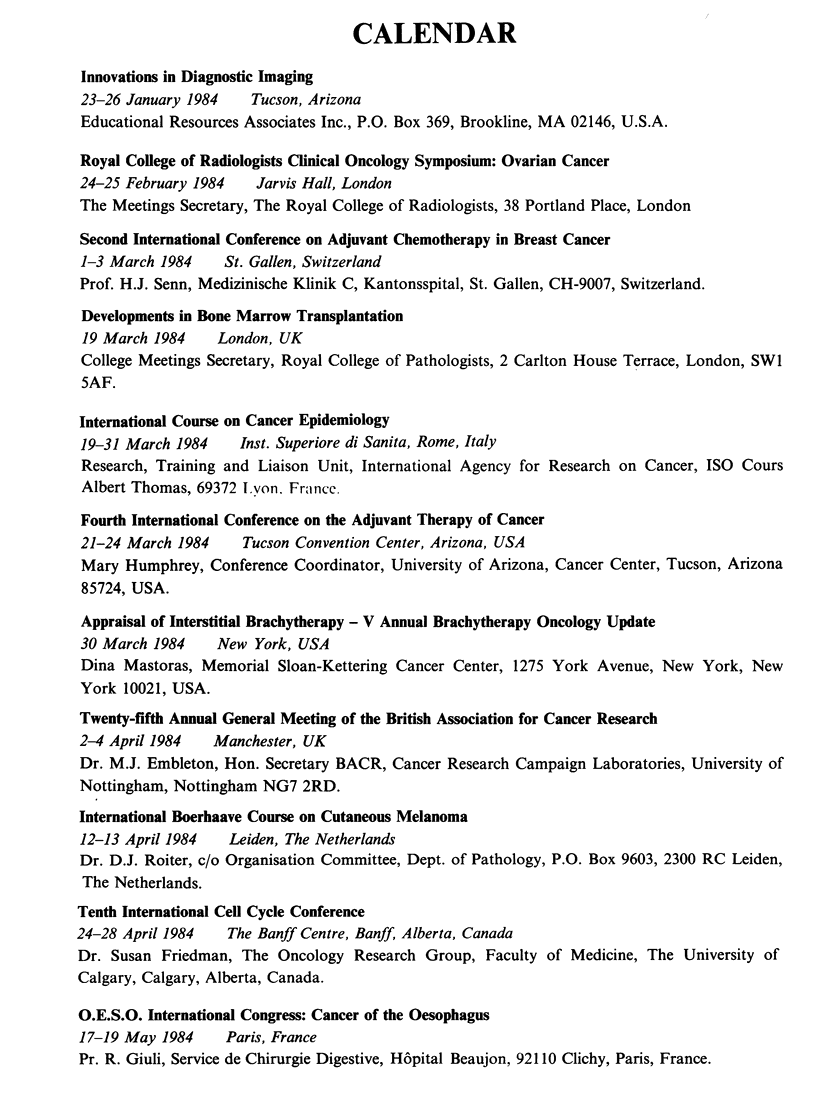# Calendar

**Published:** 1984-01

**Authors:** 


### CALENDAR

**Innovations in Diagnostic Imaging**
*23–26 January 1984     Tucson, Arizona*
Educational Resources Associates Inc., P.O. Box 369, Brookline, MA 02146, U.S.A.

**Royal College of Radiologists Clinical Oncology Symposium: Ovarian Cancer**
*24–25 February 1984     Jarvis Hall, London*
The Meetings Secretary, The Royal College of Radiologists, 38 Portland Place, London

**Second International Conference on Adjuvant Chemotherapy in Breast Cancer**
*1–3 March 1984     St. Gallen, Switzerland*
Prof. H.J. Senn, Medizinische Klinik C, Kantonsspital, St. Gallen, CH-9007, Switzerland.

**Developments in Bone Marrow Transplantation**
*19 March 1984     London, UK*
College Meetings Secretary, Royal College of Pathologists, 2 Carlton House Terrace, London, SW1 5AF.

**International Course on Cancer Epidemiology**
*19–31 March 1984     Inst. Superiore di Sanita, Rome, Italy*
Research, Training and Liaison Unit, International Agency for Research on Cancer, ISO Cours Albert Thomas, 69372 Lyon, France.

**Fourth International Conference on the Adjuvant Therapy of Cancer**
*21–24 March 1984     Tucson Convention Center, Arizona, USA*
Mary Humphrey, Conference Coordinator, University of Arizona, Cancer Center, Tucson, Arizona 85724, USA.

**Appraisal of Interstitial Brachytherapy – V Annual Brachytherapy Oncology Update**
*30 March 1984     New York, USA*
Dina Mastoras, Memorial Sloan-Kettering Cancer Center, 1275 York Avenue, New York, New York 10021, USA.

**Twenty-fifth Annual General Meeting of the British Association for Cancer Research**
*2–4 April 1984     Manchester, UK*
Dr. M.J. Embleton, Hon. Secretary BACR, Cancer Research Campaign Laboratories, University of Nottingham, Nottingham NG7 2RD.

**International Boerhaave Course on Cutaneous Melanoma**
*12–13 April 1984     Leiden, The Netherlands*
Dr. D.J. Roiter, c/o Organisation Committee, Dept. of Pathology, P.O. Box 9603, 2300 RC Leiden, The Netherlands.

**Tenth International Cell Cycle Conference**
*24–28 April 1984     The Banff Centre, Banff, Alberta, Canada*
Dr. Susan Friedman, The Oncology Research Group, Faculty of Medicine, The University of Calgary, Calgary, Alberta, Canada.

**O.E.S.O. International Congress: Cancer of the Oesophagus**
*17–19 May 1984     Paris, France*
Pr. R. Giuli, Service de Chirurgie Digestive, Hôpital Beaujon, 92110 Clichy, Paris, France.